# Supplementary material for: Is child anemia associated with early childhood development? A cross-sectional analysis of nine Demographic and Health Surveys
Source: PLoS One. 2024 Feb 28;19(2):e0298967. doi: 10.1371/journal.pone.0298967 (PMC10901303; doi:10.1371/journal.pone.0298967)
Supplement: S1 Table — (DOCX) [file pone.0298967.s001.docx]

S1 Table. Percentage of children with and without ECD data among children with any anemia and the percentage of children with and without anemia among children’s mean ECD Index.

|  |  | **Percentage of children with any anemia** | | |  | **Mean value of ECD Index** | | |
| --- | --- | --- | --- | --- | --- | --- | --- | --- |
| **Country** | **Year** | **children with ECD data** | **children without ECD data** | **p-value** |  | **children with anemia data** | **children without anemia data** | **p-value** |
| Benin | 2017–18 | 62.1 | 65.5 | 0.399 |  | 57.5 | 57.6 | 0.953 |
| Burundi | 2016–17 | 56.1 | 51.5 | 0.897 |  | 40.9 | 39.5 | 0.399 |
| Cambodia | 2014 | 43.0 | 86.0 | 0.006 |  | 72.7 | 74.3 | 0.491 |
| Haiti | 2016–17 | 57.6 | 56.1 | 0.581 |  | 64.8 | 94.8 | 0.002 |
| Jordan | 2017–18 | 26.8 | 26.1 | 0.715 |  | 70.0 | 82.0 | 0.026 |
| Maldives | 2016–17 | 45.6 | 0.0 | - |  | 92.6 | 94.4 | 0.432 |
| Rwanda | 2019–20 | 26.3 | 0.0 | - |  | 81.9 | 77.9 | 0.016 |
| Senegal | 2017 | 58.9 | 49.9 | 0.377 |  | 66.1 | 68.3 | 0.604 |
| Uganda | 2016 | 41.0 | 44.4 | 0.797 |  | 65.3 | 65.8 | 0.760 |
