# Supplementary material for: Is child anemia associated with early childhood development? A cross-sectional analysis of nine Demographic and Health Surveys
Source: PLoS One. 2024 Feb 28;19(2):e0298967. doi: 10.1371/journal.pone.0298967 (PMC10901303; doi:10.1371/journal.pone.0298967)
Supplement: S2 Table — Notes: Maternal height was not collected in Senegal 2017. Child nutritional status was not included in Jordan 2017–18 due to data quality concerns. 1 Stunted, underweight, overweight, or wasted were categorized as follows: children under 5 in the household were categorized as underweight, or normal according to the weight-for-age Z-score, categorized as stunted or normal according to the height-for-age Z-score, and categorized as wasted, normal, or overweight according to the weight-for-height Z-score in comparison to the mean on the WHO Child Growth Standards scale. (DOCX) [file pone.0298967.s002.docx]

S2 Table. Variables used in analysis.

| Variable | Definition | Categories | Binary variable constructed |  |
| --- | --- | --- | --- | --- |
| *Early learning/interaction* | | | |  |
| Early childhood education | Youngest children age 3–4 years living with interviewed mother who attend any organized learning or early childhood education program, such as a private or government facility, including kindergarten or community child care | Yes/No | Yes/No |  |
| Availability of books | Number of children’s books or picture books available for youngest child age 0–4 years living with interviewed mother | - Continuous 0–9 - 10+ | Child has 3 or more books available to them |  |
| Availability of playthings | Youngest children age 0–4 years living with interviewed mother who have toys available | - homemade toys or other toys made at home - toys from a shop or manufactured toys - household objects or objects found outside | Child has toys from at least 2 categories available to them |  |
| Support for learning | Youngest children age 0–4 years living with interviewed mother who engage in activities with household members over the age of 15 in the past 3 days | - read books to or looked at picture books with child - told stories to child - sang songs to or with child - took child outside the home, compound, yard, or enclosure - played with the child - named, counted, or drew things with child | Child engaged in any (1 or more) activities with an adult household member |  |
| Adequate care | Number of times youngest children age 0–4 years living with interviewed mother were left alone for more than an hour or left in the care of another child less than 10 years of age for more than an hour | Continuous 0–7 | Child was not left alone or in the care of another child less than 10 years of age for more than an hour at any time in the past week |  |
| *Physical Growth* | | | | |
| Nutritional status | Children under 5 in the household who were not any of the following: stunted, underweight, overweight, or wasted^1^ | Yes/No | Yes/No |  |
| *Wellness* | | | | |
| Wellness | Children under 5 in the household whose mothers report that the child had not had diarrhea, fever, or cough in the past 2 weeks | Yes/No | Yes/No |  |
| *Other covariates* | | | | |
| Age of child | Age of child in months | Continuous |  |  |
| Maternal height | Maternal height in cm | - Short maternal stature (<145 cm) - No short maternal stature (>145 cm) | Short maternal stature (Yes/No) |  |
| Maternal work status | Mother worked in the last 7 days | Yes/No | Yes/No |  |
| Maternal education | Highest educational level of the mother | - No education - Primary (ref) - Secondary - Higher |  |  |
| Paternal education | Highest educational level of the father | - No education - Primary (ref) - Secondary - Higher |  |  |
| Wealth index | Composite measure of a household’s cumulative living standard divided into quintiles. | - Lowest - Second - Middle - Fourth - Highest |  |  |
| Place of residence | Country census definitions were used to characterize rural and urban residence | Rural/urban | Rural/urban |  |
| Region | Country’s first administrative level | First administrative level designations according to country |  |  |
| Number of adults age 15 or above in the household | Number of adults age 15 or above in the household | - 2 or fewer de jure residents over age 15 - 3+ de jure residents over age 15 | 2 or fewer/3+ de jure residents over age 15 |  |
| Number of children under 5 in the household | Number of children under 5 in the household | - 2 or fewer children under age 5 - 3+ children under age 5 | 2 or fewer/3+ children under age 5 |  |
| Availability of improved sanitation and improved water source in the household | Improved sanitation includes: Include any non-shared toilet of the following types: flush/pour flush toilets to piped sewer systems and pit latrines, ventilated improved pit (VIP) latrines, and pit latrines with slabs.  Improved water source includes: piped water, protected springs, and rainwater. Households that use bottled water for drinking are classified as using an improved source only if the water they use for cooking and hand washing comes from an improved source | - No improved water source, no improved toilet - No improved water source, improved toilet - Improved water source, no improved toilet - Improved water source, improved toilet |  |  |

Notes: Maternal height was not collected in Senegal 2017. Child nutritional status was not included in Jordan 2017–18 due to data quality concerns.

^1^ Stunted, underweight, overweight, or wasted were categorized as follows: children under 5 in the household were categorized as underweight, or normal according to the weight-for-age Z-score, categorized as stunted or normal according to the height-for-age Z-score, and categorized as wasted, normal, or overweight according to the weight-for-height Z-score in comparison to the mean on the WHO Child Growth Standards scale.
