# Supplementary material for: Is child anemia associated with early childhood development? A cross-sectional analysis of nine Demographic and Health Surveys
Source: PLoS One. 2024 Feb 28;19(2):e0298967. doi: 10.1371/journal.pone.0298967 (PMC10901303; doi:10.1371/journal.pone.0298967)
Supplement: S3 Table — In the descriptive table, education was categorized as any education versus none; wealth was categorized as top 3 wealth quintiles versus the bottom 2 quintiles; and WASH was categorized as improved water and sanitation versus other. +Malnourished refers to children who are not stunted, underweight, overweight, or wasted. (DOCX) [file pone.0298967.s003.docx]

S3 Table. Descriptive statistics of variables, stratified by anemia status for each country.

| **Survey** | **Anemia status** | **Early childhood education** | **Availability of books** | **Availability of playthings** | **Adequate care** | **Support for learning** | **No illness in past 2 weeks** | **Not malnourished^+^** | **Mother’s education (any)** | **Mother working** | **Father's education (any)** | **Mother's height <145cm** | **3+ adults in household** | **3+ children under 5 in household** | **Wealth (top 3 quintiles)** | **WASH (improved water source & toilet)** | **Residence (urban)** | **Unweighted N** |
| --- | --- | --- | --- | --- | --- | --- | --- | --- | --- | --- | --- | --- | --- | --- | --- | --- | --- | --- |
|  |  |  |  |  |  |  |  |  |  |  |  |  |  |  |  |  |  |  |
|  |  |  |  |  |  |  |  |  |  |  |  |  |  |  |  |  |  |  |
| Benin 2017-18 | None, mild anemia | 21.3 | 4.0 | 62.2 | 65.9 | 43.4 | 73.7 | 67.4 | 81.8 | 86.0 | 81.5 | 0.8 | 50.9 | 31.0 | 46.3 | 28.8 | 41.4 | 1412 |
|  | Severe, moderate anemia | 11.8 | 1.0 | 52.9 | 59.5 | 39.0 | 66.9 | 55.5 | 86.5 | 83.4 | 81.1 | 1.3 | 55.3 | 38.6 | 25.1 | 14.3 | 26.3 | 756 |
|  | All | 18.0 | 3.0 | 59.0 | 63.7 | 41.9 | 71.3 | 63.3 | 83.4 | 85.1 | 81.4 | 1.0 | 52.4 | 33.6 | 38.9 | 23.8 | 36.2 | 2168 |
| Burundi 2016-17 | None, mild anemia | 10.4 | 0.3 | 38.5 | 54.2 | 61.6 | 46.8 | 41.1 | 60.4 | 88.2 | 63.0 | 3.8 | 31.2 | 16.7 | 41.5 | 48.4 | 10.3 | 1628 |
|  | Severe, moderate anemia | 1.5 | 0.2 | 32.5 | 50.3 | 59.8 | 34.3 | 22.8 | 63.4 | 88.6 | 62.0 | 5.6 | 25.0 | 17.5 | 22.0 | 35.4 | 4.3 | 699 |
|  | All | 7.5 | 0.2 | 36.5 | 52.9 | 61.0 | 42.7 | 35.0 | 61.4 | 88.3 | 62.7 | 4.4 | 29.1 | 17.0 | 35.0 | 44.1 | 8.3 | 2327 |
| Cambodia 2014 | None, mild anemia | 14.9 | 5.2 | 41.1 | 84.6 | 46.5 | 68.3 | 54.7 | 43.3 | 70.5 | 58.5 | 5.4 | 47.5 | 8.7 | 34.9 | 38.9 | 13.1 | 1404 |
|  | Severe, moderate anemia | 7.1 | 3.5 | 39.1 | 87.1 | 39.9 | 61.3 | 41.3 | 45.6 | 71.2 | 55.9 | 6.4 | 42.2 | 4.8 | 26.1 | 28.4 | 7.9 | 223 |
|  | All | 13.9 | 5.0 | 40.8 | 84.9 | 45.7 | 67.4 | 53.0 | 43.6 | 70.5 | 58.2 | 5.5 | 46.9 | 8.2 | 33.8 | 37.6 | 12.5 | 1627 |
| Haiti 2016-17 | None, mild anemia | 64.5 | 14.9 | 58.3 | 76.2 | 55.3 | 39.4 | 77.4 | 63.3 | 58.3 | 81.2 | 0.5 | 52.5 | 15.1 | 36.7 | 45.1 | 32.9 | 1058 |
|  | Severe, moderate anemia | 55.4 | 12.0 | 52.5 | 72.7 | 49.9 | 38.6 | 69.6 | 61.1 | 55.6 | 79.5 | 0.9 | 45.6 | 13.7 | 30.3 | 39.0 | 37.6 | 354 |
|  | All | 62.0 | 14.1 | 56.7 | 75.3 | 53.9 | 39.2 | 75.2 | 62.7 | 57.6 | 80.8 | 0.6 | 50.6 | 14.7 | 35.0 | 43.4 | 34.1 | 1412 |
| Jordan 2017-18 | None, mild anemia | 11.7 | 21.9 | 76.7 | 83.0 | 91.8 | 78.7 | 49.8 | 94.9 | 12.0 | 91.3 | 0.4 | 25.0 | 18.8 | 29.1 | 99.9 | 89.4 | 1842 |
|  | Severe, moderate anemia | 8.0 | 11.8 | 72.9 | 88.5 | 86.7 | 81.5 | 57.5 | 94.1 | 7.1 | 84.9 | 0.0 | 24.4 | 23.9 | 21.1 | 100.0 | 86.6 | 154 |
|  | All | 11.4 | 21.1 | 76.4 | 83.4 | 91.4 | 78.9 | 50.4 | 94.8 | 11.6 | 90.8 | 0.4 | 25.0 | 19.2 | 28.4 | 99.9 | 89.2 | 1996 |
| Maldives 2016-17 | None, mild anemia | 79.0 | 75.9 | 55.5 | 87.6 | 97.9 | 65.0 | 70.8 | 77.8 | 34.9 | 83.1 | 8.9 | 73.1 | 18.3 | 26.4 | 98.9 | 25.6 | 769 |
|  | Severe, moderate anemia | 73.3 | 60.2 | 54.4 | 79.4 | 95.9 | 50.6 | 73.1 | 83.4 | 36.5 | 89.2 | 7.2 | 84.5 | 32.1 | 40.0 | 96.9 | 38.8 | 137 |
|  | All | 78.0 | 73.2 | 55.3 | 86.3 | 97.5 | 62.6 | 71.2 | 78.8 | 35.2 | 84.1 | 8.6 | 75.0 | 20.6 | 28.7 | 98.6 | 27.8 | 906 |
| Rwanda 2019-20 | None, mild anemia | 30.6 | 2.7 | 43.3 | 66.7 | 37.1 | 68.8 | 63.1 | 35.0 | 77.4 | 44.7 | 2.1 | 34.1 | 11.1 | 38.8 | 59.4 | 17.4 | 1315 |
|  | Severe, moderate anemia | 32.3 | 2.1 | 35.4 | 54.9 | 39.2 | 56.8 | 50.0 | 24.4 | 77.8 | 44.2 | 1.5 | 30.1 | 6.8 | 19.8 | 57.6 | 10.2 | 133 |
|  | All | 30.7 | 2.6 | 42.6 | 65.7 | 37.3 | 67.8 | 61.9 | 34.1 | 77.4 | 44.6 | 2.0 | 33.7 | 10.7 | 37.1 | 59.3 | 16.7 | 1448 |
|  |  |  |  |  |  |  |  |  |  |  |  |  |  |  |  |  |  |  |
| Senegal 2017 | None, mild anemia | 21.9 | 1.8 | 25.7 | 70.6 | 30.7 | 72.8 | 78.1 | 75.1 | 58.2 | 86.5 | 49.5 | 85.2 | 57.2 | 38.0 | 62.4 | 41.6 | 2771 |
|  | Severe, moderate anemia | 13.5 | 0.3 | 17.6 | 65.1 | 24.3 | 70.2 | 67.0 | 81.1 | 53.8 | 88.8 | 52.0 | 85.7 | 60.0 | 20.0 | 47.9 | 23.7 | 1273 |
|  | All | 19.5 | 1.4 | 23.4 | 69.1 | 28.9 | 72.0 | 75.0 | 76.8 | 57.0 | 87.1 | 50.2 | 85.4 | 58.0 | 32.9 | 58.3 | 36.5 | 4044 |
| Uganda 2016 | None, mild anemia | 45.3 | 4.4 | 59.8 | 58.9 | 54.2 | 44.1 | 71.8 | 39.4 | 81.7 | 59.6 | 0.9 | 31.1 | 19.6 | 41.9 | 36.2 | 22.7 | 1265 |
|  | Severe, moderate anemia | 28.1 | 1.0 | 48.3 | 47.6 | 44.6 | 34.2 | 57.7 | 37.2 | 76.9 | 54.4 | 1.2 | 29.8 | 25.5 | 19.9 | 19.8 | 10.1 | 345 |
|  | All | 41.9 | 3.7 | 57.5 | 56.6 | 52.3 | 42.1 | 69.0 | 39.0 | 80.7 | 58.6 | 1.0 | 30.9 | 20.8 | 37.5 | 33.0 | 20.2 | 1610 |

In the descriptive table, education was categorized as any education versus none; wealth was categorized as top 3 wealth quintiles versus the bottom 2 quintiles; and WASH was categorized as improved water and sanitation versus other.

^+^Malnourished refers to children who are not stunted, underweight, overweight, or wasted.
