# Supplementary material for: Is child anemia associated with early childhood development? A cross-sectional analysis of nine Demographic and Health Surveys
Source: PLoS One. 2024 Feb 28;19(2):e0298967. doi: 10.1371/journal.pone.0298967 (PMC10901303; doi:10.1371/journal.pone.0298967)
Supplement: S4 Table — In the table, education was categorized as any education versus none; wealth was categorized as top 3 wealth quintiles versus the bottom 2 quintiles; and WASH as categorized as improved water and sanitation versus other. +Malnourished refers to children who are not stunted, underweight, overweight, or wasted. (DOCX) [file pone.0298967.s004.docx]

| **Survey** | **No illness in past 2 weeks** | **Not malnourished^+^** | **Moderate/severe anemia** | **Early childhood education** | **Availability of books** | **Availability of playthings** | **Adequate care** | **Support for learning** | **Mother’s education (any)** | **Mother working** | **Father's education (any)** | **Mother's height <145cm** | **3+ adults in household** | **3+ children under 5 in household** | **Wealth (top 3 quintiles)** | **WASH (improved water source & toilet)** | **Residence (urban)** |
| --- | --- | --- | --- | --- | --- | --- | --- | --- | --- | --- | --- | --- | --- | --- | --- | --- | --- |
|  |  |  |  |  |  |  |  |  |  |  |  |  |  |  |  |  |  |
|  |  |  |  |  |  |  |  |  |  |  |  |  |  |  |  |  |  |
| Benin 2017-18 | 1.01 | 1.01 | 1.02 | 1.23 | 1.14 | 1.06 | 1.02 | 1.15 | 1.06 | 1.01 | 1.04 | 1.00 | 1.18 | 1.16 | 1.48 | 1.41 | 1.20 |
| Burundi 2016-17 | 1.01 | 1.03 | 1.05 | 1.05 | 1.00 | 1.05 | 1.04 | 1.02 | 1.03 | 1.03 | 1.03 | 1.01 | 1.07 | 1.01 | 1.33 | 1.16 | 1.23 |
| Cambodia 2014 | 1.00 | 1.01 | 1.01 | 1.07 | 1.08 | 1.07 | 1.00 | 1.08 | 1.09 | 1.02 | 1.11 | 1.01 | 1.07 | 1.04 | 2.02 | 1.90 | 1.31 |
| Haiti 2016-17 | 1.00 | 1.01 | 1.01 | 1.19 | 1.14 | 1.12 | 1.03 | 1.19 | 1.09 | 1.03 | 1.08 | 1.01 | 1.04 | 1.06 | 2.07 | 1.66 | 1.53 |
| Jordan 2017-18 | 1.00 | 1.00 | 1.00 | 1.08 | 1.07 | 1.08 | 1.01 | 1.09 | 1.04 | 1.03 | 1.06 | 1.01 | 1.01 | 1.04 | 1.08 | 1.00 | 1.04 |
| Maldives 2016-17 | 1.02 | 1.01 | 1.01 | 1.05 | 1.09 | 1.04 | 1.01 | 1.02 | 1.12 | 1.04 | 1.08 | 1.01 | 1.08 | 1.10 | 2.77 | 1.01 | 2.78 |
| Rwanda 2019-20 | 1.01 | 1.01 | 1.01 | 1.03 | 1.04 | 1.04 | 1.01 | 1.05 | 1.07 | 1.03 | 1.03 | 1.01 | 1.06 | 1.01 | 1.38 | 1.17 | 1.24 |
| Senegal 2017 | 1.00 | 1.02 | 1.01 | 1.14 | 1.06 | 1.05 | 1.01 | 1.13 | 1.06 | 1.01 | 1.04 | 1.01 | 1.14 | 1.18 | 2.03 | 1.55 | 1.83 |
| Uganda 2016 | 1.01 | 1.02 | 1.02 | 1.09 | 1.06 | 1.04 | 1.01 | 1.09 | 1.18 | 1.02 | 1.08 | 1.01 | 1.02 | 1.03 | 1.55 | 1.50 | 1.27 |

S4 Table. Variance Inflation Factors (VIF) for covariates included in the regression for each country.

In the table, education was categorized as any education versus none; wealth was categorized as top 3 wealth quintiles versus the bottom 2 quintiles; and WASH as categorized as improved water and sanitation versus other

^+^Malnourished refers to children who are not stunted, underweight, overweight, or wasted.
