# Supplementary material for: Is child anemia associated with early childhood development? A cross-sectional analysis of nine Demographic and Health Surveys
Source: PLoS One. 2024 Feb 28;19(2):e0298967. doi: 10.1371/journal.pone.0298967 (PMC10901303; doi:10.1371/journal.pone.0298967)
Supplement: S8 Table — AOR = adjusted odds ratio, LB = Lower bound of 95% confidence interval; UB = upper bound of 95% confidence interval. Models were adjusted for region. In Jordan child nutritional status was not included and in Senegal maternal height was not included. Blank cells indicate that no coefficients were produced in the model because of small sample sizes. + Malnourished refers to children who are not stunted, underweight, overweight, or wasted. (DOCX) [file pone.0298967.s008.docx]

S8 Table. Adjusted odds ratios for on-track for social-emotional development among children age 36–59 months across all countries.

|  | **Benin 2017-18** | | | | **Burundi 2016-17** | | | | **Cambodia 2014** | | | | **Haiti 2016-17** | | | | **Jordan 2017-18** | | | |
| --- | --- | --- | --- | --- | --- | --- | --- | --- | --- | --- | --- | --- | --- | --- | --- | --- | --- | --- | --- | --- |
| **Variable** | **AOR** | **LB** | **UB** | **p-value** | **AOR** | **LB** | **UB** | **p-value** | **AOR** | **LB** | **UB** | **p-value** | **AOR** | **LB** | **UB** | **p-value** | **AOR** | **LB** | **UB** | **p-value** |
| Severe or moderate anemia | 1.00 | 0.99 | 1.00 | 0.023 | 1.00 | 1.00 | 1.00 | 0.469 | 1.00 | 0.99 | 1.00 | 0.261 | 1.00 | 1.00 | 1.01 | 0.465 | 1.00 | 1.00 | 1.01 | 0.428 |
| Early child education | 0.89 | 0.60 | 1.32 | 0.578 | 1.20 | 0.70 | 2.04 | 0.504 | 0.85 | 0.53 | 1.37 | 0.507 | 0.83 | 0.51 | 1.36 | 0.468 | 0.70 | 0.39 | 1.27 | 0.245 |
| Availability of books | 0.43 | 0.20 | 0.95 | 0.036 | 0.27 | 0.06 | 1.23 | 0.090 | 0.76 | 0.38 | 1.53 | 0.445 | 1.28 | 0.67 | 2.46 | 0.458 | 1.66 | 1.00 | 2.74 | 0.050 |
| Availability of playthings | 1.00 | 0.75 | 1.33 | 0.997 | 0.78 | 0.60 | 1.02 | 0.070 | 1.12 | 0.82 | 1.53 | 0.478 | 1.16 | 0.79 | 1.70 | 0.458 | 0.72 | 0.44 | 1.17 | 0.186 |
| Adequate care | 1.33 | 1.01 | 1.76 | 0.044 | 1.31 | 1.03 | 1.66 | 0.025 | 1.45 | 0.92 | 2.28 | 0.107 | 1.75 | 1.13 | 2.72 | 0.013 | 0.62 | 0.42 | 0.92 | 0.018 |
| Support for learning | 1.08 | 1.01 | 1.16 | 0.032 | 1.04 | 0.97 | 1.13 | 0.258 | 1.07 | 0.98 | 1.16 | 0.114 | 0.97 | 0.87 | 1.10 | 0.667 | 0.99 | 0.86 | 1.14 | 0.909 |
| Age in months (36-59) | 1.01 | 0.99 | 1.03 | 0.397 | 1.01 | 0.99 | 1.03 | 0.187 | 1.00 | 0.98 | 1.02 | 0.793 | 1.00 | 0.98 | 1.03 | 0.915 | 0.99 | 0.96 | 1.01 | 0.343 |
| No illness past 2 weeks | 1.19 | 0.91 | 1.55 | 0.194 | 1.18 | 0.93 | 1.49 | 0.174 | 0.87 | 0.63 | 1.22 | 0.423 | 1.22 | 0.83 | 1.79 | 0.301 | 1.01 | 0.70 | 1.47 | 0.938 |
| Not malnourished^+^ | 1.05 | 0.80 | 1.37 | 0.717 | 1.02 | 0.78 | 1.33 | 0.910 | 1.50 | 1.11 | 2.02 | 0.008 | 0.90 | 0.57 | 1.44 | 0.666 |  |  |  |  |
| Mother's education level |  |  |  |  |  |  |  |  |  |  |  |  |  |  |  |  |  |  |  |  |
| None | 0.80 | 0.55 | 1.17 | 0.251 | 0.98 | 0.76 | 1.27 | 0.894 | 1.52 | 1.00 | 2.33 | 0.051 | 1.76 | 1.02 | 3.04 | 0.041 | 1.09 | 0.37 | 3.21 | 0.872 |
| Primary | 1.00 |  |  |  | 1.00 |  |  |  | 1.00 |  |  |  | 1.00 |  |  |  | 1.00 |  |  |  |
| Secondary | 0.83 | 0.49 | 1.38 | 0.469 | 1.21 | 0.68 | 2.15 | 0.512 | 1.26 | 0.86 | 1.85 | 0.235 | 1.08 | 0.63 | 1.84 | 0.784 | 0.80 | 0.37 | 1.72 | 0.560 |
| Higher | 18.55 | 2.05 | 168.25 | 0.010 | 5.42 | 1.19 | 24.81 | 0.029 | 1.05 | 0.30 | 3.58 | 0.944 | 2.12 | 0.28 | 15.90 | 0.464 | 0.76 | 0.33 | 1.76 | 0.527 |
| Mother working | 0.81 | 0.53 | 1.24 | 0.331 | 1.36 | 0.91 | 2.05 | 0.133 | 0.91 | 0.61 | 1.36 | 0.645 | 0.98 | 0.65 | 1.46 | 0.916 | 1.31 | 0.73 | 2.35 | 0.361 |
| Mother's height < 145 cm | 1.95 | 0.52 | 7.23 | 0.318 | 0.69 | 0.41 | 1.15 | 0.158 | 1.38 | 0.71 | 2.68 | 0.342 | 0.57 | 0.06 | 5.09 | 0.612 | 0.46 | 0.12 | 1.76 | 0.255 |
| Father's education level |  |  |  |  |  |  |  |  |  |  |  |  |  |  |  |  |  |  |  |  |
| None | 1.07 | 0.75 | 1.53 | 0.716 | 1.34 | 1.05 | 1.71 | 0.021 | 1.02 | 0.59 | 1.77 | 0.940 | 0.66 | 0.40 | 1.07 | 0.093 | 1.63 | 0.61 | 4.36 | 0.329 |
| Primary | 1.00 |  |  |  | 1.00 |  |  |  | 1.00 |  |  |  | 1.00 |  |  |  | 1.00 |  |  |  |
| Secondary | 1.18 | 0.81 | 1.71 | 0.397 | 0.99 | 0.60 | 1.62 | 0.956 | 1.12 | 0.77 | 1.61 | 0.552 | 0.77 | 0.44 | 1.35 | 0.361 | 1.79 | 1.00 | 3.19 | 0.050 |
| Higher | 1.10 | 0.50 | 2.40 | 0.811 | 0.64 | 0.21 | 1.88 | 0.411 | 1.08 | 0.44 | 2.63 | 0.870 | 1.55 | 0.59 | 4.11 | 0.374 | 2.09 | 1.08 | 4.03 | 0.028 |
| 3+ adults in household | 1.30 | 0.97 | 1.74 | 0.083 | 1.18 | 0.92 | 1.51 | 0.202 | 0.96 | 0.70 | 1.32 | 0.813 | 1.18 | 0.78 | 1.77 | 0.431 | 1.40 | 0.93 | 2.09 | 0.104 |
| 3+ children under 5 in household | 0.81 | 0.60 | 1.08 | 0.149 | 0.62 | 0.46 | 0.82 | 0.001 | 1.30 | 0.68 | 2.48 | 0.420 | 1.42 | 0.75 | 2.70 | 0.281 | 1.07 | 0.66 | 1.74 | 0.783 |
| WASH |  |  |  |  |  |  |  |  |  |  |  |  |  |  |  |  |  |  |  |  |
| Water-, toilet- | 1.00 |  |  |  | 1.00 |  |  |  | 1.00 |  |  |  | 1.00 |  |  |  |  |  |  |  |
| Water-, toilet+ | 0.80 | 0.38 | 1.72 | 0.574 | 1.07 | 0.61 | 1.86 | 0.825 | 1.14 | 0.61 | 2.14 | 0.671 | 1.17 | 0.48 | 2.89 | 0.726 |  |  |  |  |
| Water+, toilet- | 1.04 | 0.75 | 1.44 | 0.807 | 0.78 | 0.51 | 1.21 | 0.270 | 1.16 | 0.74 | 1.82 | 0.512 | 0.84 | 0.48 | 1.44 | 0.516 |  |  |  |  |
| Water+, toilet+ | 1.78 | 1.11 | 2.85 | 0.017 | 1.02 | 0.65 | 1.61 | 0.924 | 0.90 | 0.55 | 1.47 | 0.679 | 1.41 | 0.69 | 2.87 | 0.342 |  |  |  |  |
| Wealth quintile |  |  |  |  |  |  |  |  |  |  |  |  |  |  |  |  |  |  |  |  |
| Poorest | 1.00 |  |  |  | 1.00 |  |  |  | 1.00 |  |  |  | 1.00 |  |  |  | 1.00 |  |  |  |
| Poorer | 1.14 | 0.76 | 1.70 | 0.524 | 0.93 | 0.63 | 1.36 | 0.693 | 1.19 | 0.76 | 1.87 | 0.437 | 1.13 | 0.64 | 1.98 | 0.673 | 0.70 | 0.46 | 1.07 | 0.103 |
| Middle | 0.89 | 0.59 | 1.35 | 0.585 | 0.74 | 0.51 | 1.06 | 0.103 | 0.69 | 0.41 | 1.17 | 0.168 | 0.86 | 0.41 | 1.81 | 0.683 | 0.52 | 0.33 | 0.84 | 0.007 |
| Richer | 0.94 | 0.59 | 1.50 | 0.793 | 0.82 | 0.55 | 1.21 | 0.307 | 0.51 | 0.28 | 0.94 | 0.031 | 0.78 | 0.31 | 1.93 | 0.588 | 0.44 | 0.23 | 0.82 | 0.009 |
| Richest | 0.47 | 0.25 | 0.89 | 0.020 | 0.72 | 0.42 | 1.23 | 0.232 | 0.70 | 0.32 | 1.51 | 0.364 | 0.74 | 0.25 | 2.23 | 0.596 | 0.57 | 0.28 | 1.15 | 0.117 |
| Residence |  |  |  |  |  |  |  |  |  |  |  |  |  |  |  |  |  |  |  |  |
| Urban | 1.00 |  |  |  | 1.00 |  |  |  | 1.00 |  |  |  | 1.00 |  |  |  | 1.00 |  |  |  |
| Rural | 1.12 | 0.78 | 1.62 | 0.544 | 1.37 | 0.83 | 2.29 | 0.221 | 0.74 | 0.45 | 1.20 | 0.218 | 0.93 | 0.49 | 1.74 | 0.816 | 0.99 | 0.64 | 1.53 | 0.958 |

S8 Table—*Continued*

|  | **Maldives 2016-17** | | | | **Rwanda 2019-20** | | | | **Senegal 2017** | | | | **Uganda 2016** | | | |
| --- | --- | --- | --- | --- | --- | --- | --- | --- | --- | --- | --- | --- | --- | --- | --- | --- |
| **Variable** | **AOR** | **LB** | **UB** | **p-value** | **AOR** | **LB** | **UB** | **p-value** | **AOR** | **LB** | **UB** | **p-value** | **AOR** | **LB** | **UB** | **p-value** |
| Severe or moderate anemia | 1.00 | 0.99 | 1.01 | 0.565 | 1.00 | 0.99 | 1.01 | 0.741 | 1.00 | 1.00 | 1.00 | 0.421 | 1.00 | 1.00 | 1.01 | 0.173 |
| Early child education | 1.16 | 0.61 | 2.22 | 0.653 | 0.59 | 0.30 | 1.16 | 0.126 | 1.62 | 1.07 | 2.45 | 0.022 | 1.12 | 0.78 | 1.61 | 0.554 |
| Availability of books | 1.26 | 0.67 | 2.40 | 0.471 | 0.41 | 0.06 | 2.71 | 0.356 | 2.10 | 0.59 | 7.44 | 0.250 | 0.90 | 0.32 | 2.53 | 0.840 |
| Availability of playthings | 1.18 | 0.62 | 2.26 | 0.605 | 1.44 | 0.76 | 2.74 | 0.260 | 0.68 | 0.49 | 0.94 | 0.019 | 0.70 | 0.49 | 1.00 | 0.052 |
| Adequate care | 0.71 | 0.29 | 1.74 | 0.457 | 3.26 | 1.65 | 6.44 | 0.001 | 0.75 | 0.58 | 0.96 | 0.023 | 1.28 | 0.91 | 1.81 | 0.156 |
| Support for learning | 0.99 | 0.65 | 1.49 | 0.946 | 0.90 | 0.70 | 1.16 | 0.424 | 0.77 | 0.70 | 0.84 | 0.000 | 1.10 | 1.01 | 1.20 | 0.032 |
| Age in months (36-59) | 0.97 | 0.93 | 1.02 | 0.267 | 1.01 | 0.97 | 1.06 | 0.513 | 1.01 | 1.00 | 1.03 | 0.132 | 0.99 | 0.97 | 1.01 | 0.416 |
| No illness past 2 weeks | 0.67 | 0.32 | 1.41 | 0.286 | 2.16 | 1.13 | 4.09 | 0.019 | 1.03 | 0.80 | 1.32 | 0.819 | 1.55 | 1.12 | 2.14 | 0.008 |
| Not malnourished^+^ | 0.55 | 0.26 | 1.14 | 0.107 | 0.67 | 0.34 | 1.32 | 0.247 | 1.11 | 0.87 | 1.40 | 0.401 | 1.01 | 0.70 | 1.45 | 0.972 |
| Mother's education level |  |  |  |  |  |  |  |  |  |  |  |  |  |  |  |  |
| None | 3.35 | 0.47 | 24.08 | 0.227 | 1.66 | 0.80 | 3.42 | 0.173 | 0.76 | 0.54 | 1.09 | 0.140 | 1.33 | 0.82 | 2.15 | 0.241 |
| Primary | 1.00 |  |  |  | 1.00 |  |  |  | 1.00 |  |  |  | 1.00 |  |  |  |
| Secondary | 1.93 | 0.77 | 4.85 | 0.162 | 1.90 | 0.51 | 7.08 | 0.338 | 0.58 | 0.30 | 1.15 | 0.117 | 0.93 | 0.59 | 1.49 | 0.771 |
| Higher | 0.78 | 0.18 | 3.39 | 0.745 | 1.00 |  |  |  | 1.26 | 0.38 | 4.15 | 0.702 | 2.78 | 1.01 | 7.68 | 0.049 |
| Mother working | 1.17 | 0.63 | 2.16 | 0.616 | 1.35 | 0.58 | 3.11 | 0.485 | 1.06 | 0.82 | 1.38 | 0.631 | 1.09 | 0.69 | 1.71 | 0.718 |
| Mother's height < 145 cm | 2.17 | 0.83 | 5.66 | 0.114 | 2.32 | 0.28 | 19.03 | 0.432 |  |  |  |  | 0.46 | 0.12 | 1.76 | 0.259 |
| Father's education level |  |  |  |  |  |  |  |  |  |  |  |  |  |  |  |  |
| None | 0.61 | 0.18 | 2.06 | 0.427 | 0.58 | 0.26 | 1.26 | 0.168 | 0.93 | 0.66 | 1.30 | 0.662 | 1.76 | 0.76 | 4.04 | 0.186 |
| Primary | 1.00 |  |  |  | 1.00 |  |  |  | 1.00 |  |  |  | 1.00 |  |  |  |
| Secondary | 1.02 | 0.40 | 2.60 | 0.970 |  |  |  |  | 0.89 | 0.54 | 1.47 | 0.653 | 1.34 | 0.86 | 2.08 | 0.191 |
| Higher | 1.25 | 0.46 | 3.38 | 0.656 |  |  |  |  | 1.59 | 0.65 | 3.87 | 0.310 | 1.38 | 0.76 | 2.49 | 0.292 |
| 3+ adults in household | 0.99 | 0.51 | 1.95 | 0.985 | 0.88 | 0.43 | 1.82 | 0.735 | 0.87 | 0.55 | 1.38 | 0.556 | 1.01 | 0.72 | 1.42 | 0.945 |
| 3+ children under 5 in household | 0.58 | 0.28 | 1.19 | 0.136 | 1.01 | 0.35 | 2.97 | 0.982 | 0.89 | 0.66 | 1.21 | 0.463 | 1.33 | 0.88 | 2.01 | 0.178 |
| WASH |  |  |  |  |  |  |  |  |  |  |  |  |  |  |  |  |
| Water-, toilet- |  |  |  |  | 1.00 |  |  |  | 1.00 |  |  |  | 1.00 |  |  |  |
| Water-, toilet+ |  |  |  |  | 0.70 | 0.15 | 3.32 | 0.653 | 0.64 | 0.37 | 1.11 | 0.115 | 2.52 | 0.95 | 6.67 | 0.063 |
| Water+, toilet- |  |  |  |  | 0.58 | 0.15 | 2.27 | 0.435 | 0.85 | 0.57 | 1.28 | 0.430 | 1.43 | 0.86 | 2.38 | 0.163 |
| Water+, toilet+ |  |  |  |  | 0.47 | 0.13 | 1.73 | 0.256 | 1.02 | 0.66 | 1.57 | 0.925 | 2.00 | 0.97 | 4.11 | 0.059 |
| Wealth quintile |  |  |  |  |  |  |  |  |  |  |  |  |  |  |  |  |
| Poorest | 1.00 |  |  |  | 1.00 |  |  |  | 1.00 |  |  |  | 1.00 |  |  |  |
| Poorer | 1.39 | 0.66 | 2.97 | 0.386 | 0.83 | 0.37 | 1.85 | 0.642 | 1.02 | 0.74 | 1.41 | 0.883 | 0.79 | 0.49 | 1.29 | 0.355 |
| Middle | 1.01 | 0.48 | 2.14 | 0.974 | 2.74 | 0.92 | 8.17 | 0.070 | 0.86 | 0.56 | 1.34 | 0.510 | 0.81 | 0.50 | 1.31 | 0.384 |
| Richer | 1.84 | 0.47 | 7.20 | 0.381 | 1.14 | 0.46 | 2.82 | 0.771 | 0.86 | 0.49 | 1.51 | 0.598 | 0.49 | 0.28 | 0.88 | 0.016 |
| Richest | 0.42 | 0.06 | 3.10 | 0.396 | 3.17 | 0.74 | 13.50 | 0.118 | 0.69 | 0.30 | 1.63 | 0.399 | 0.51 | 0.23 | 1.15 | 0.107 |
| Residence |  |  |  |  |  |  |  |  |  |  |  |  |  |  |  |  |
| Urban | 1.00 |  |  |  | 1.00 |  |  |  | 1.00 |  |  |  | 1.00 |  |  |  |
| Rural | 0.88 | 0.18 | 4.30 | 0.873 | 0.42 | 0.14 | 1.30 | 0.134 | 1.05 | 0.74 | 1.51 | 0.769 | 0.81 | 0.45 | 1.45 | 0.472 |

AOR=adjusted odds ratio, LB=Lower bound of 95% confidence interval; UB=upper bound of 95% confidence interval.

Models were adjusted for region. In Jordan child nutritional status was not included and in Senegal maternal height was not included.

Blank cells indicate that no coefficients were produced in the model because of small sample sizes.

^+^ Malnourished refers to children who are not stunted, underweight, overweight, or wasted
